# Light-activated cell identification and sorting (LACIS) for selection of edited clones on a nanofluidic device

**DOI:** 10.1038/s42003-018-0034-6

**Published:** 2018-05-03

**Authors:** Annamaria Mocciaro, Theodore L. Roth, Hayley M. Bennett, Magali Soumillon, Abhik Shah, Joseph Hiatt, Kevin Chapman, Alexander Marson, Gregory Lavieu

**Affiliations:** 1 Berkeley Lights, Inc, 5858 Horton Street #320, Emeryville, CA 94608 USA; 2 UCSF Department of Microbiology and Immunology, University of California, San Francisco, CA 94143 USA; 3 Chan Zuckerberg Biohub, San Francisco, CA 94158 USA; 4 Diabetes Center, University of California, San Francisco, CA 94143 USA; 5 Innovative Genomics Institute, University of California, Berkeley, CA 94720 USA; 6 Department of Medicine, University of California, San Francisco, CA 94143 USA; 7 UCSF Helen Diller Family Comprehensive Cancer Center, University of California, San Francisco, CA 94158 USA; 8 Institut Curie, INSERM U932–Immunity and Cancer, Paris, 75005, France

## Abstract

Despite improvements in the CRISPR molecular toolbox, identifying and purifying properly edited clones remains slow, laborious, and low-yield. Here, we establish a method to enable clonal isolation, selection, and expansion of properly edited cells, using OptoElectroPositioning technology for single-cell manipulation on a nanofluidic device. Briefly, after electroporation of primary T cells with *CXCR4*-targeting Cas9 ribonucleoproteins, single T cells are isolated on a chip and expanded into colonies. Phenotypic consequences of editing are rapidly assessed on-chip with cell-surface staining for CXCR4. Furthermore, individual colonies are identified based on their specific genotype. Each colony is split and sequentially exported for on-target sequencing and further off-chip clonal expansion of the validated clones. Using this method, single-clone editing efficiencies, including the rate of mono- and bi-allelic indels or precise nucleotide replacements, can be assessed within 10 days from Cas9 ribonucleoprotein introduction in cells.

## Abstract (web summary)

Annamaria Mocciaro et al. present LACIS, a method for identifying and selecting gene-edited clones using a microfluidic device. The authors apply LACIS to primary T cells after CRISPR-Cas9 editing of *CXCR4* and show that selection of edited clones was possible within 10 days from initiation of gene editing.

## Introduction

Cell engineering through gene editing is fundamentally a two-step bioprocess. In the upstream step, delivery of genome editing machinery to the cell type of interest generates efficient and specific edits. The downstream step involves identification and selection of the cells that have been properly edited.

Cas9-mediated gene editing is a powerful tool to engineer cell lines and primary cells^1–3^. The method enables precise correction or introduction of mutations within an endogenous genomic locus through co-delivery of a DNA template for homology-directed repair (HDR). There are widespread efforts to use this approach in clinically relevant systems to model genetic disorders^4^ and for gene therapy to correct disease-driving mutations^5^.

Many research and therapeutic applications are currently limited by the low efficiency of precise HDR-based editing. Even with improved delivery of Cas9, some targeted cells remain unedited. In addition, Cas9-mediated DNA breaks are repaired frequently by non-homologous end joining (NHEJ) mechanisms that can introduce varying insertion and deletion mutations (indels) at the cut site resulting in undesirable editing outcomes^6,7^. Precise editing is complicated further because two copies of somatic alleles are present in the diploid genome. Therefore, in a given cell, HDR-mediated editing might occur only on one allele while the other allele is either unedited or imprecisely edited by NHEJ-mediated repair. Progress has been made to enhance the efficiency of HDR-based editing^8^, however, a technology to identify cells with desired mono- or bi-allelic edits is urgently needed to realize the full potential of CRISPR.

Selection of edited cell clones currently relies on limiting dilution or fluorescence-activated cell sorting (FACS)-based single-cell sorting to isolate single cells. When genome editing induces a phenotypic alteration that is detectable by fluorescence (i.e., cell-surface expression of a target that can be non-lethally assessed with fluorescently labeled antibody), FACS provides a method of enriching edited cells^9^, narrowing the number of clones to propagate and analyze. However, when the desired edit is phenotypically silent, a larger number of clones need to be selected for sequencing to ensure that at least one of them has been properly edited. Moreover, even when high-purity single-cell sorting can be achieved, viability after sorting is often low to moderate, especially for cell types that are particularly sensitive to hydrodynamic stress or low-density culture conditions (e.g., primary cells or pluripotent stem cell lines). As a consequence, investigators often need to isolate a large number of clones and then proceed with tedious and time-consuming efforts to expand all of them individually. Each clonal line must then be assessed by sequencing to find those that bear the desired edits. Generating validated clonal lines can require several weeks. Therefore, the development of a method that allows screening of edited cells and minimizes cell manipulation and hands-on culturing would constitute a significant addition to the current genome engineering toolbox.

Here, we present proof-of-concept data highlighting the abilities of a new platform that integrates mechanical, fluidic, electrical, and optical modules to enable single-cell manipulation, clonal expansion, and phenotypic analysis in nanoliter volumes. The platform takes advantage of the OptoSelect^TM^ technology (described below), which allows light-controlled manipulation of single cells^10–12^.

The advantages of the OptoSelect technology include the capacity for massive parallel cell manipulation; on-chip clonal expansion through absolute control of CO_2_, temperature, and media perfusion; on-chip fluorescence-based phenotypic assessment; and sequential export of clones of interest for downstream processing. Every step of the workflow is computed and the process is highly automated such that it can be operated in a largely (>90%) hands-off manner. This new platform has allowed us to develop a method that facilitates both identification and selection of properly edited cells, including human primary T cells, as shown in the experiments presented in this study.

Here, we interrogate individual T-cell colonies on-chip after electroporation. Up to 50% of single T cells loaded on chip proliferate into a colony and fewer than 20% of the cells electroporated with *CXCR4* editing reagents have detectable CXCR4 cell-surface labeling (vs. 80–90% CXCR4+ in control T cells electroporated with scrambled  guide RNA (gRNA). After export of selected clones from the chip, >50% of the exported clones are able to proliferate and can be used in downstream applications. Concomitant genotypic assessment of the exported clones through on-target sequencing reveals that approximately 5% of the putative edited candidates have bi-allelic HDR-based edits. Therefore, the proposed method enables the identification and selection of precisely edited clones within 10 days from Cas9–RNP introduction in cells.

## Results

### Overview of the platform technology

**Fig. 1:**
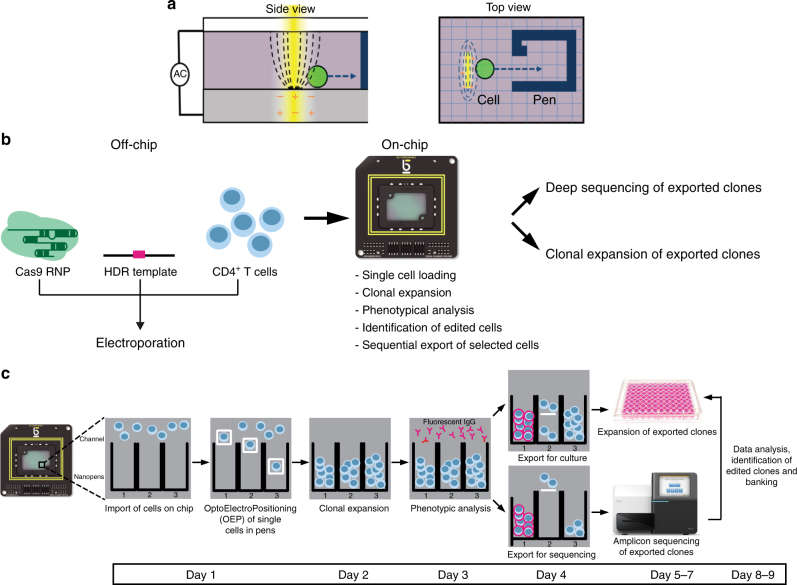
Method to identify and select edited cell with high precision. **a** Schematic side (left panel) and top (right panel) views of the chip, depicting the OEP principle. A single-cell (green) is moved inside a NanoPen (blue solid lines, blue arrow) through OEP (yellow bar, dashed lines). **b**, **c** Schematic representation of the LACIS workflow. T-cell electroporation is performed off-chip, while clonal expansion, phenotype assessment, and export are performed on-chip. Each colony is split and exported. The first half of the colony is exported and further expanded through off-chip culture, while the remaining half is exported for validation through amplicon sequencing of the *CXCR4* locus. After on-target validation, the desired clones are selected for further expansion and banking

The data presented in this work were generated using a platform that enables single-cell manipulation in a nanofluidic device, using OptoElectroPositioning (OEP). The OEP principle is based on the generation of light-induced dielectrophoresis (DEP), an electrical gradient force. The nanofluidic device (the OptoSelect^TM^ chip) consists of a transparent electrode on a silicon substrate with a fluidic chamber sandwiched between the two. The substrate is fabricated with an array of photosensitive transistors. When focused light hits the transistors and a voltage is applied, a non-uniform electric field is generated. This imparts a negative DEP force that repels particles (including cells) using light-induced OEP (Fig. 1a). In the absence of targeted light, no force is generated. When light is shined on the photoconductive material, DEP force is generated and cells trapped inside light “cages” can be moved across the chamber. In addition, NanoPens™ are integrated into the chip to isolate cells from each other, enabling on-chip culture of well-separated colonies emanating from single cells. The chip is placed on a 3-axis robotic stage and an upright microscope mounted on top of the stage allows image collection of the entire chip area, to monitor cell growth, morphology, and to perform phenotype analyses. After characterization, selected clones can be exported off the chip for further processing. The export is the reverse of the import process, where desired cells are moved using OEP from single NanoPens into the main channel and flushed into a target well of a 96-well plate positioned inside a CO2- and temperature-controlled incubator (Fig. 1c).

### On-chip clonal expansion and phenotyping of edited T cells

**Fig. 2:**
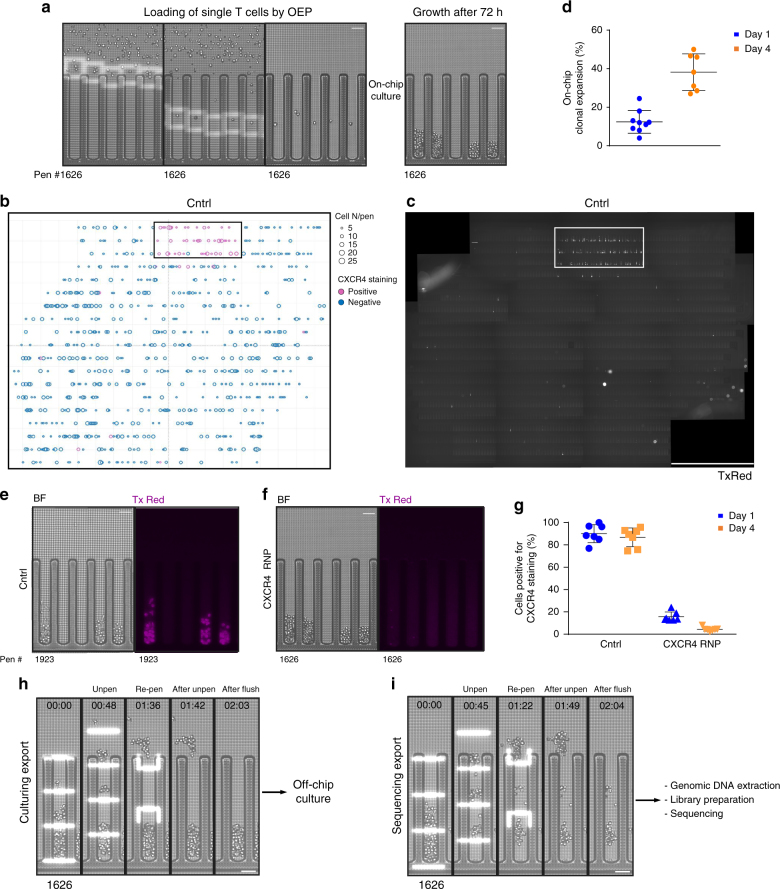
On-chip clone expansion, identification, and selection. **a** Electroporated cells located in the channel are automatically identified and singles are captured within light cages (white squares) and positioned into NanoPens, then cultured for 3 days. Bar = 50 μm. **b** Schematic representation of OCCE and CXCR4 staining as a function of on-chip positioning. Each circle represents a single colony within a NanoPen, identified by XY coordinates. The diameter of the circle is proportional to the colony size. Clones positive for CXCR4 are depicted as magenta circles. The heavy box indicates the field of view reserved for control cells. **c** Composite image of the entire chip in the TxRed channel, showing CXCR4 staining. Eighteen fields of view were assembled together. White rectangle shows control cells loaded within a single field of view. The other 17 fields of view contain clones electroporated with CXCR4 Cas9 RNPs. Bar = 3.3 mm. **d** Dot plot of OCCE in chips loaded 1 (blue circles) or 4 (orange circles) days after electroporation. Bars represent mean ± SD of OCCE in 9 or 7 chips per condition, respectively (>300 clones per chip). **e**, **f** On-chip phenotype assessment with fluorescent anti-CXCR4 antibody. Left panel, control cells. Right panel, edited candidates, negative for CXCR4 staining. **g** Quantification of CXCR4 staining for control and edited cells loaded 1 (blue) or 4 (orange) days after electroporation. Bars indicate mean ± SD of CXCR4 staining in seven chips per condition (>300 clones analyzed per chip). **h**, **i** Representative images of the split export process: during Culture Export, the first half of the colony is unpenned using OEP (white bars), pushed into the channel, and exported into a well of a 96-well plate. Remaining cells are pushed back into the NanoPen to ensure clonality. For sequencing export, the second half of the colony is exported into a second 96-well plate for on-target sequencing. Numbers in the upper portion of the panels indicate the duration of a single export (in minutes). Bar = 50 μm

As previously described, human primary T cells were transfected with Cas9 ribonucleproteins (RNPs) targeting *CXCR4*, a gene encoding a surface receptor that acts as a co-receptor for HIV^9^. The RNP complex was mixed with a short ssDNA oligonucleotide HDR template designed to replace 12 nucleotides within *CXCR4 *(Fig. 1b) and impair cell-surface expression. We previously reported up to ~20% HDR efficiency at this locus^9^ based on deep sequencing analysis of a bulk population of edited cells. However, bulk sequencing of alleles from a cell population cannot distinguish the portion of mono- and bi-allelic knock-ins at the single-cell level. To obtain both phenotypic and genotypic data from individual edited clones, T cells were imported onto the chip for one (day 1) or 4 days (day 4) after electroporation with CXCR4 Cas9 RNPs (Fig. 2a). We assessed editing efficiency at these two time-points to identify further timeline compression options. After loading, flow was stopped to keep cells immobile within the main channel, which distributes media to multiple NanoPens (up to 3500/chip) through diffusion. Single cells were automatically selected and trapped into light cages that enable single-cell positioning within the NanoPens, in 17 out of the 18 fields of view that are visualized on the chip (Fig. 2a–c, Supplementary Movie 1). Non-penned cells remaining within the channel were flushed out of the chip. Importantly, we performed a second import with T cells electroporated with RNPs containing a scrambled control gRNA that does not target any locus in the human genome, positioning them in the remaining field of view (Fig. 2b, c, Supplementary Movie 2). After 3 days of culture, during which fresh media was perfused into the main channel, we assessed on-chip clonal expansion. We first identified the pens that were initially loaded with single cells (to ensure clonality) and counted the number of pens that contained >6 cells after 3 days of culture. We established that, across multiple chips, approximately 15 ± 6% of single cells loaded at day 1 (Fig. 2d, blue circles), or 40 ± 9% of single cells loaded at day 4 (Fig. 2d, orange circles) formed a colony. The size of the individual colonies was heterogeneous (Fig. 2b, solid circles). The average doubling time was about 24 ± 2 h (day 1) and 18 ± 2 h (day 4) over 3 days of growth (Supplementary Fig. 1A). The lower off-chip clonal expansion (OCCE) and the longer doubling time of cells loaded at day 1 are likely due to higher cell death caused by the introduction of the RNP complex and the ssDNA in the first 48 h after electroporation. These data strongly suggest that manipulation by OEP does not impair cell viability, and that diffusion of nutrients from the channel to the NanoPens maintains cell growth at expected levels. Importantly, we used NanoPens that were initially empty to track putative on-chip cross-contamination (cell transferred from one pen to another). Fewer than 2% of initially empty pens acquired visible cells within the 3 days of culture in both conditions, indicating >98% on-chip clonality (Supplementary Fig. 1B). This rare cross-contamination that was observed might be explained by the high motility of activated T cells.

Next, we established an on-chip phenotypic assay to identify clones that had undergone successful *CXCR4* editing. Fluorescently labeled anti-CXCR4 antibody was imported into the chip, and media flow was interrupted to allow diffusion of the antibody into the NanoPens. After 45 min of incubation, the chip was continuously flushed for 30 min with fresh media, to remove excess free antibody. Fluorescent images of the entire chip were taken (Fig. 2c, e, f) and the number of colonies positive for CXCR4 surface expression was quantified. Among the colonies formed by control cells across all chips, roughly 95% (day 1) and 85% (day 4) of clones were positive for CXCR4 (Fig. 2e, g). Strikingly, for CXCR4-edited cells loaded 1 day after electroporation, only 20% of the colonies showed presence of CXCR4 on the cell surface. In cells from healthy donors loaded 4 days post-electroporation, the number of colonies positive for CXCR4 staining dropped to around 5% (Fig. 2f, g). Importantly, each single pen was assessed for colony formation and fluorescence signal and a report was automatically generated to identify the NanoPens containing the clones of interest (Fig. 2b, c).

### On-target validation and expansion of exported clones

Among all the putative edited clones that were automatically identified we selected the clones with the highest OCCE and created a short list of candidates to export for on-target validation through high-throughput sequencing. In each experimental replicate, 48 clones were exported from each chip, and three chips per experiment were used for subsequent validation. Our goal was to validate as early as possible the desirable clones in order to avoid wasting hands-on culturing efforts on clones that were not properly edited. To achieve this, we developed a pipeline that enabled a “split export” for clones of interest. Briefly, for each selected colony, roughly half of the cells were moved from the NanoPen into the channel via light bars (Fig. 2h, Supplementary Movie 3). Unpenned cells (>5 cells/colony) were flushed out and collected in a defined well of a 96-well plate kept in a CO_2_- and temperature-controlled incubator for further off-chip culture. We termed this step “culture export.” Cells were exported from 48 NanoPens of each chip in this manner. We inserted 48 control blank exports (from empty NanoPens) between each clonal export to assess cross-contamination between wells introduced during and after export. Following culture export, media was replaced with export buffer and remaining cells from each NanoPen’s colony were serially transferred in the main channel and flushed out within a small volume of buffer into a corresponding well of a 96-well PCR plate kept at 4 °C. We termed this step “sequencing export” (Fig. 2i, Supplementary Movie 4). Efficiency of the export process, defined as the fraction of NanoPens from which >1 cell was transferred to the channel, was >80% (Supplementary Fig. 1C). The modest reduction in export efficiency was due to cells that clustered within the NanoPens and diminished the effect of the OEP force on cell movability.

**Fig. 3:**
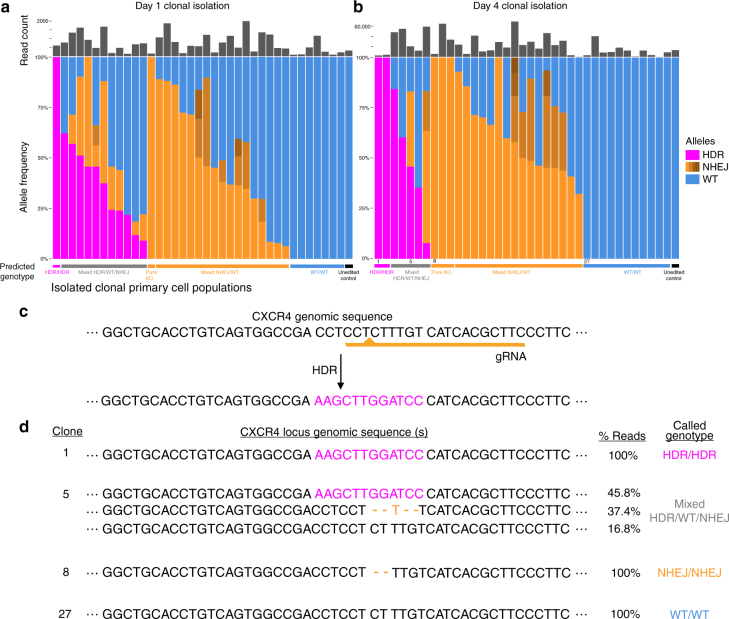
Off-chip sequencing of editing outcomes in individual clones. **a**, **b** Proportions of reads mapping to HDR (magenta), NHEJ (orange), or WT (blue) editing outcomes in each individual clone isolated and sequenced from cells loaded on-chip either 1 day **a** post-electroporation or 4 days **b** post-electroporation. The total read count from each clone in the sequencing run is displayed above the allele frequency. **c**, **d** Clones with many different genotypes can be identified, including those that integrated the HDR template (**c**) on both alleles (100% HDR, Clone 1), as well as clones with the same NHEJ edit on both alleles (Clone 8 with a two base-pair deletion), or mixed genotypes with more than two alleles present (Clone 5, potentially due to CRISPR editing continuing to happen after an initial round of division post single-cell isolation)

Immediately after the sequencing export, collected cells (>5 cells per colony) were lysed and prepared for on-target sequencing of the *CXCR4* locus (Supplementary Fig. 2A, B). The sequencing reads from each individual clone were then aligned to the *CXCR4* WT sequence (blue), the predicted HDR sequence (magenta), or neither (called as a NHEJ due to introduced indel or point mutations, orange) (Supplementary Fig. 2C). Aggregating all the alleles found in cells from clones isolated on-chip on either day 1 or day 4 post electroporation allowed for a genotype to be assigned to each clone (Fig. 3a, b). In one healthy human blood donor, clones could be identified that possessed a variety of genotypes, from no edits at all (homozygous reference allele), to mixed alleles of NHEJ-introduced indels, to mono-allelic HDR (with either reference sequence or indels on the other allele), to bi-allelic HDR (HDR/HDR) (Fig. 3a, b). Of note, not all *CXCR4*-edited clones identified with loss of CXCR4 surface expression had 100% editing at the targeted CXCR4 locus, potentially due to Cas9 steric hindering CXCR4 transcription but not inducing a noticeable cut; large deletions unable to be identified by amplicon sequencing; or other unknown factors. More than two individual alleles were found in some clones, potentially due to editing events occurring after the first cell division (i.e., four alleles now present that could be edited), or cross-contamination between wells during culture, export, or NGS library preparation (Supplementary Fig. 3).

Sequencing a portion of a clonal population while maintaining an ongoing culture of cells from the same colony allowed for clones to be identified based on their genotype, such as bi-allelic HDR (Fig. 3c). Selected examples of genotypes of clones isolated day 4 post-electroporation demonstrate the ability to identify such bi-allelic HDR integrations (Fig. 3d). To assess the fidelity of the off-chip sequencing and confirm the short ssDNA HDR template was not causing sequencing artifacts, we sequenced several individual unedited control clones (black, unedited controls electroporated with a scrambled gRNA-based Cas9 RNP as well as the same HDR template as used for *CXCR4*-edited cells) that had been loaded in a pre-determined area of the chip and exported (Fig. 2b, c). As expected, >97% of control clones showed no genomic alteration in the targeted *CXCR4* locus (homozygous reference genotype, Fig. 3a, b). Overall, sequencing revealed that bi-allelically edited HDR clones could be identified while maintaining a live culture of the same clones.

Independently, we then assessed the post-export viability within the culture export plate. Exported clones were maintained for an additional week in culture, then images of the export plates were taken and colony formation was quantified (Supplementary Fig. 4A). Depending on the export conditions, up to 80% of the exported clones were able to survive and expand, with an average of 50% of viability across all chips. Notably, we observed some variability in colony survival rates after export. In one replicate of the experiment, the off-chip post-export viability was below 20% (Supplementary Fig. 4B). In that experiment, the number of cells exported from each NanoPen was on average <5. We then refined our analysis, and we observed a strong correlation between the off-chip colony survival rate and the number of cells exported from each NanoPen (Supplementary Fig. 4C). We concluded that, with current protocols, at least ten cells needed to be exported for further off-chip clonal expansion in order to ensure >50% post-export viability. Overall, this criterion was satisfied by >75% of the exported clones (Supplementary Fig. 4D).

With approximately 5% bi-allelic HDR editing at the *CXCR4* locus and >50% post-export viability, our results suggest that as few as 100 clones could be screened for on-target sequencing validation to ensure that at least 1–2 precisely edited primary human T-cell clones are collected after culture export and will survive clonal expansion. This method is immediately relevant to identify and bank accurately edited clones of human primary cells.

## Discussion

Here, we demonstrate that the light-activated cell identification and sorting (LACIS) method is well-suited to rapidly isolate clones that have been properly edited with precision. Compared to other methods, LACIS provides multiple advantages: this workflow removes the wasteful hands-on cell culture effort on undesired clones that are not properly edited. In addition, desired clones are identified quickly (<10 days), allowing for increased iterations and faster bioprocess optimization. Exporting larger numbers of cells per clone directly improves viability and expansion of the selected clones, and therefore contributes to increase the overall process efficiency. Importantly, this workflow can be almost fully automated, which will enable significantly enhanced scale relative to current protocols.

In this study, we focused on primary human T-cell editing. We showed that the current capacity of the chip enables the identification of bi-allelically HDR-edited T cells, which at the targeted *CXCR4* locus was approximately 5% of edited cells. Therefore, even for a low-efficiency editing target, the presented workflow is advantageous and should guarantee successful selection of cells with the desired genotype, whether or not edited cells can be phenotypically selected.

To our knowledge, the present study is the first demonstration of a broadly applicable method that will enable selection of edited cells based on genotype and/or phenotype. The initial use of FACS enabled only a modest fourfold enrichment of a certain cell sub-type based on one fluorescent criteria^13^, but now—nearly 50 years later—enrichment can reach thousands of fold and allows multi-parametric analysis of heterogeneous cell populations. This offers some perspective for future improvements in experimental throughput that will require innovative design of the chip to enable massive parallel genotyping and phenotyping throughout the entire chip (>3000 clones) within each run.

In our study, we primarily focused on the genotypic validation of edited clones through targeted DNA sequencing. However, further development of our platform would enable to analyze mutation-induced perturbations at the whole-transcriptome level through RNA sequencing, thus introducing an additional way of linking genotype and phenotype, which is critical to understand disease genetics and characterize new therapeutic targets.

Recent improvements in high-throughput sequencing—especially barcoding and low input (5–20 cells) processing—are driving cost reductions that will enable larger scale characterization of edited cells while also assessing off-target effects when necessary^14–16^. This promises to greatly facilitate and improve the manufacturing of edited cell lines for the scientific and medical community. In addition, our flexible platform could enhance other gene-editing workflows. For instance, our pipeline could facilitate the study of genetic disorders through the generation of heterozygous or homozygous model cell lines ( Embryonic Stem Cells (ESCs) or induced Pluripotent Stem Cells (iPSCs) bearing knock-in disease mutation^17–19^.

Much remains to be done to truly revolutionize the process of generating precisely edited clonal populations. For instance, culture and export of adherent cell lines need to be enabled, since they constitute many relevant models for disease. Chemical modification of the surface coating of the chip, combined with treatment with cell dissociation reagents, may enable efficient manipulation of adherent cells in the future. This should also enhance efficient manipulation of all cell types that have the tendency to form clusters, which can affect OEP efficiency. In addition, gene and cell therapies currently require dealing with very large numbers of cells. Therefore, modifications of the platform’s design will be needed to make it fully compatible with these applications. Automation, new microfluidic layouts and integration of relevant technologies will soon enable efficient high-throughput sorting of cells based on genotype with absolute precision.

## Methods

### Human T-cell isolation and culture

Primary human T-cell culture and RNP editing has been previously described^9^. Briefly, PBMCs were isolated using SepMate tubes (STEMCELL) per manufacturer’s instructions from blood from healthy human donors under a UCSF CRB approved protocol. CD3+ T cells were negatively isolated from PBMCs using an EasySep (STEMCELL) negative magnetic isolation kit per manufacturer’s protocol. T cells were stimulated with plate bound CD3 (10 µg mL^–1^, Tonbo Biosciences, clone UCHT1) and soluble CD28 (5 µg mL^–1^, Tonbo Biosciences, clone CD28.2) antibodies at 1 million cells per 1 mL of RPMI media with 10% FBS. After electroporation, T cells were stimulated with CD3/CD28 dynabeads (Cell Therapy Systems, 1:1 bead to cell ratio) and 20 U mL^–1^ of IL-2 (UCSF Pharmacy) again at 1 million cells per mL of media until import onto the Optoselect chip.

### Cas9 RNPs electroporation

A two-component gRNA system was used- crRNAs targeting either *CXCR4* or no human genomic sequence (for gRNAs sequences, see Supplementary Data 1) were synthesized (Dharmacon) and resuspended in 10 mM Tris-HCl pH 7.4 with 150 mM KCl to a final concentration of 160 µM. tracrRNA was similarly synthesized and resuspended. The crRNA and tracrRNA were mixed 1:1 by volume and incubated for 30 min at 37 °C to produce 80 µM gRNA. Forty micrometer SpCas9 (QB3 Macrolab) was added at 1:1 by volume to the gRNA (a 1:2 molar ratio of Cas9 to gRNA) and incubated for 15 min at 37 °C to yield a 20 µM RNP. RNPs were prepared immediately before electroporation into T cells. A short ssDNA HDR template (ssODN) to insert a defined 12 bp sequence into *CXCR4* was chemically synthesized (IDT) and resuspended in nuclease-free H_2_O at 100 µM. The same *CXCR4* targeting HDR template was used for both *CXCR4* and scrambled gRNAs (for ssDNA sequence, see Supplementary Data 1). Two days following stimulation, T cells were harvested and resuspended in P3 electroporation buffer (Lonza) at a concentration of 1 million cells per 20 µL of buffer. Five microliter of RNP (100 pmols) were added to 20 µL of cells (1 million T cells) along with 1 µL of HDR template (100 pmols) were mixed and electroporated in a single well of a Lonza nucleofection cuvette on a Lonza Nucleofector 4D X-Unit device using pulse program EH-115. Immediately following electroporation, 80 µL of pre-warmed culture media were added directly to the cuvette and the cells were allowed to rest in a 5% CO_2_ 37 °C incubator for 15 min in the cuvettes before being stimulated and transferred out for further culture (see human T-cell isolation and culture).

### Preparation of cell suspension for penning in Optoselect chip

T cells were cultured for 1 day or 4 days after electroporation in culture media [RPMI–1640 (Gibco) supplemented with 2 mmol L^–1^ Glutamax (Gibco), 10% (vol/vol) FBS (Seradigm), 2% Human AB serum (ZenBio) and 50 IU mL^–1^ IL-2 (R&D Systems), in the presence of Anti-CD3/CD28 Dynabeads (Gibco)]. Prior to loading onto the chip, cells were resuspended in culture media supplemented with 10 ng mL^–1^ IL-7 and IL-15 (PeProTech) at a final density of 5e6 cells mL^–1^.

### Conditions for automated cell penning

Experiments were conducted on Berkeley Lights, Inc. platforms and Optoselect chips. After priming, chips were washed twice with de-ionized water and flushed six times with culture media. Cells were imported onto the chips and loaded as single cells into NanoPens using OEP with the following parameters—nominal voltage: 4.5 V; frequency: 1000 kHz; cage shape: square; cage speed: 8 µm s^–1^; cage line width: 10 µm. Loading temperature was set to 36 °C. Brightfield images of each chip were acquired automatically at the end of the loading process and a BerkeleyLights, Inc. proprietary algorithm was used to detect and count cells by pen.

### Culturing conditions and cell expansion quantification

Chips were maintained at a temperature of 36 °C during culture. CO_2_-buffered culture media was perfused through the chip at a flow rate of 0.01 µL s^–1^. For primary cell growth assessment and automated counting, Brightfield images of the chips were taken at distinct time points to quantify on-chip clonal expansion (OCCE), defined as the percentage of NanoPens containing a single-cell that grew into a colony of six or more cells after 72 h of culture. Cross-contamination across each chip was determined as the percentage of initially empty pens that acquired cells during culture.

### On-chip T-cell staining

Cell surface staining was performed with αCXCR4–PE (12G5; BioLegend). The antibody was imported into the chip at 1:250 dilution in culture media and incubated for 45 min at 36 °C. After staining, chips were perfused for 30 min with culture media media, to remove the excess antibody, and then images were acquired in Brightfield (25 ms) and Texas Red (1000 ms) channels.

### Split export of edited clones

Three to 4 days after loading, clones containing >10 cells that showed negative staining for CXCR4 were sequentially exported for off-chip culturing and genotyping. Forty-eight clones and 48 blanks were exported per chip. In the first step of the split export (culturing export), roughly half of each clone (5–20 cells) was transferred from the NanoPen to the channel using light bars generated by OEP, with the following parameters—nominal voltage: 4.5 V; frequency: 1000 kHz; bar speed: 5 µm s^–1^; bar line width: 10 µm. Export temperature was set to 36 °C, export was performed in culture media and cells were flushed in a 20 µL package volume into a barcoded round-bottom, tissue culture treated 96-well plate containing 100 µL of culture media supplemented with 10 ng mL^–1^ IL-7 and IL-15 per well. The plate was kept in an incubator at 36 °C and 5% CO_2_ for the entire duration of the export. After 7 days of culture, images of the plates in the Brightfiled channel were collected with Cytation 3 Cell Imaging Multi-Mode Reader (BioTek), and wells where cells grew into sizeable clones were counted.

For the second step of the split export (genotyping export), culture media was replaced with export buffer [PBS (Gibco), 5 mg mL^–1^ BSA (Fisher Scientific), 0.1% Pluronic F-127 (Life Tech)] by flushing the chip ten times before starting the export. Then, the remaining cells from the previously exported pens were transferred to the channel by OEP with the following parameters—nominal voltage: 5 V; frequency: 1000 kHz; bar speed: 5 µm s^–1^; bar line width: 10 µm. Export temperature was set to 36 °C, export was performed in export buffer and cells were flushed in a 5 µL package volume into a barcoded 96-well PCR plate (Eppendorf) containing 20 µL of mineral oil (Sigma–Aldrich) and 5 µL of Proteinase K buffer [(10 mM Tris-HCl pH 8, 0.1 M NaCl, 1 mM EDTA, 200 µg mL^–1^ proteinase K (Ambion AM2546)] per well. The PCR plate was maintained at 4 °C for the entire duration of the export.

### Sample processing for next-generation sequencing

Genomic DNA was extracted from exported clones by incubating in Proteinase K buffer (0.1 M NaCl, 10 mM Tris-HCl pH 8.0, 1 mM EDTA) for 30 min at 55 °C, then for 20 min at 80 °C to inactivate Proteinase K. The genomic region around the CRISPR/Cas9 target site for *CXCR4* gene was amplified by PCR with primers positioned outside of the HDR repair template sequence (positioned to avoid amplification of exogenous template) for ten cycles using KAPA HiFi Hotstart ReadyMix (Kapa Biosystems, KR0370) according to the manufacturer’s protocol (PCR primers listed in Supplementary Data 1). Primers contained inline sample-specific barcodes. Barcoded samples from each plate were pooled to concentrate and remove mineral oil using Zymo DNA Clean and Concentrator Column (Zymo research, D4004). Excess PCR primers were removed by incubating with Exonuclease I (NEB, M0293S) in 1 × exonuclease reaction buffer (NEB, B0293S) for 1 h at 37 °C, followed by enzyme inactivation for 20 min at 80 °C. Amplicon pools were re-amplified by PCR for 15 cycles using a universal primer to add the sequencing adaptor and secondary barcodes to allow parallel sequencing of multiple amplicon pools (Primer sequences are listed in Supplementary Data 1). PCR products of the expected size were isolated with Select-A-Size DNA Clean and Concentrator (Zymo research, D4080) as sequencing libraries. Pooled barcoded libraries were sequenced with 300 bp paired-end reads on a MiSeq (Illumina) instrument using the 300 cycles v3 reagent kit (Illumina).

### Sequencing data analysis and HDR/indel identification

All computational and statistical analysis were performed using Python 2.7 and Unix-based software tools. Quality of paired-end sequencing reads (R1 and R2 fastq files) was assessed using FastQC (http://www.bioinformatics.babraham.ac.uk/projects/fastqc). Reads with sample-specific inline barcodes were demultiplexed using our home-brew python script for FASTQ files splitting. Reads were then mapped on both the wild-type sequence and the expected HDR-edited sequence of *CXCR4* using bwa version 0.7.15^17^ with default parameters. Alignments files were sorted and indexed using samtools version 1.3.1^18,19^. Variants were called using freebayes version 1.0.2^20^, a Bayesian haplotype-based polymorphism discovery tool. Genotypes were determined for each colony based on the number of reads matching either the wild-type sequence, the HDR sequence or containing variants to these two sequences with a quality above 30. Python scripts implementing the demultiplexing, alignment, and genotyping are available from the authors upon request.

### Optoselect technology and chip overview

The OptoSelect^TM^ platform takes advantage of the OptoElectroPositioning (OEP^TM^) technology, which enables light-controlled cell manipulation. OEP is enabled by the generation of a dielectrophoretic force (DEP), which occurs when a polarizable particle is suspended in a non-uniform electric field.

The proprietary OptoSelect nanofluidic device consists of a top transparent electrode and a bottom silicon substrate with a fluidic chamber in between. The substrate is fabricated with an array of photosensitive transistors. When light shines on the transistors, and if voltage is applied, a non-uniform electric field is locally generated in the fluidic channel. This imparts a negative DEP force that repels particles (including cells) using light-induced OEP. In the absence of targeted light, no force is generated; when light is shined on the photoconductive material, DEP force is generated and particles trapped inside light “cages” can be moved across the chamber. The chip contains a main fluidic channel and 3500 individual NanoPens chambers, which hold a 0.5 nL volume each. Media is perfused through the channel by a fluidic system, bringing nutrients to the NanoPens and carrying away waste, with the movement of nutrients and waste between the channel and NanoPens occurring via diffusion. Cells are loaded into the channel through an import needle, from a sample tube or from a well plate, and using light cages they are moved into the NanoPens at a speed of 5–15 µm s^–1^ (Fig. 1a). Single cells loaded into pens are isolated from each other, and perfusion of CO2-buffered media through the chip during culturing at 36 °C enables the in-pen expansion of clones over time. The chip is placed on a 3-axis robotic stage and an upright microscope mounted on top of the stage allows image collection of the entire chip area at 4 × or 10 × magnification in brightfield and fluorescent channels, to monitor cell growth, morphology, and to perform phenotypical analyses. After characterization, selected clones can be exported off the chip for further processing. The export is the reverse of the import process, where desired cells are moved using OEP from single NanoPens into the main channel and flushed into a target well of a 96-well plate positioned inside a CO2- and temperature-controlled incubator.

The imaging system can provide both brightfield and fluorescent imaging. A 360 nm LED source is used to illuminate the background and a 400–700 nm white light lamp combined with a digital micromirror device (DMD) is used to structure light in desired patterns for light actuated dielectrophoresis (DEP). The system uses an upright microscope with an automated lens changer to image at 4 × and 10 × magnifications and a linear cube slider to collect fluorescent images in wavelengths corresponding to Cy5, FITC, and TxRED fluorescent channels. More information are available at https://www.berkeleylights.com/contact–us/.

### Data availability

The custom Python script used for this study is available for download at https://github.com/abhik/crispr–analysis. Sequencing data have been deposited at The National Center for Biotechnology Information (NCBI) under BioProject number PRJNA444104 (SRA Study: SRP136206) and are available at https://www.ncbi.nlm.nih.gov/Traces/study/?acc = SRP136206.

## Electronic supplementary material

Supplementary Information

Description of additional Supplementary Infomation

Supplementary Data 1

Supplementary Movie 1

Supplementary Movie 2

Supplementary Movie 3

Supplementary Movie 4
