# Supplementary material for: Light-activated cell identification and sorting (LACIS) for selection of edited clones on a nanofluidic device
Source: Commun Biol. 2018 May 3;1:41. doi: 10.1038/s42003-018-0034-6 (PMC6123811; doi:10.1038/s42003-018-0034-6)
Supplement: Supplementary file 1 — Supplementary Information [file 42003_2018_34_MOESM1_ESM.pdf]

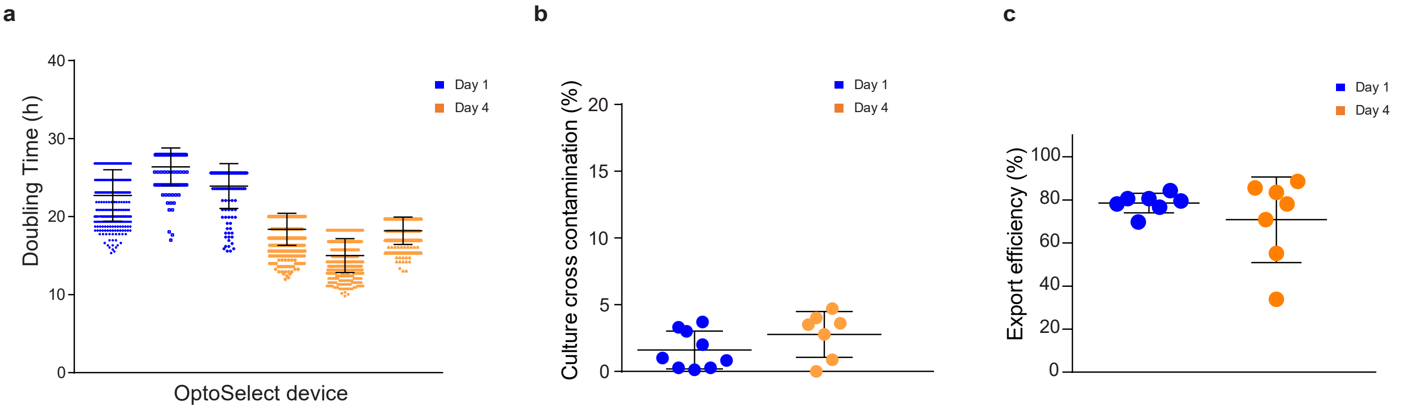

Supplementary Figure 1: Assessment of on-chip clonal expansion, cross-contamination and unpenning efficiency. a Dot plot of T-cell doubling times in 6 representative individual Optoselect devices, loaded 1 day (Day 1, blue) or 4 days (Day 4, orange) after electroporation. Bars indicate means ±SD of doubling times of >300 NanoPens analyzed per chip. **b** Dot Plot of the percentage of NanoPens that were originally empty and acquired unwanted cells over 3 days of on-chip culture. Bars indicate means ±SD of 9 or 7 chips loaded 1 day (Day 1, blue circles) or 4 days (Day 4, orange circles) after electroporation, respectively, in three independent experiments (>100 NanoPens analyzed per chip). **c** Export efficiency of clones in chips loaded 1 (blue circles) or 4 (orange circles) days after electroporation. Bars indicate means ±SD of export efficiency in 7 chips per condition (48 clones exported per chip).

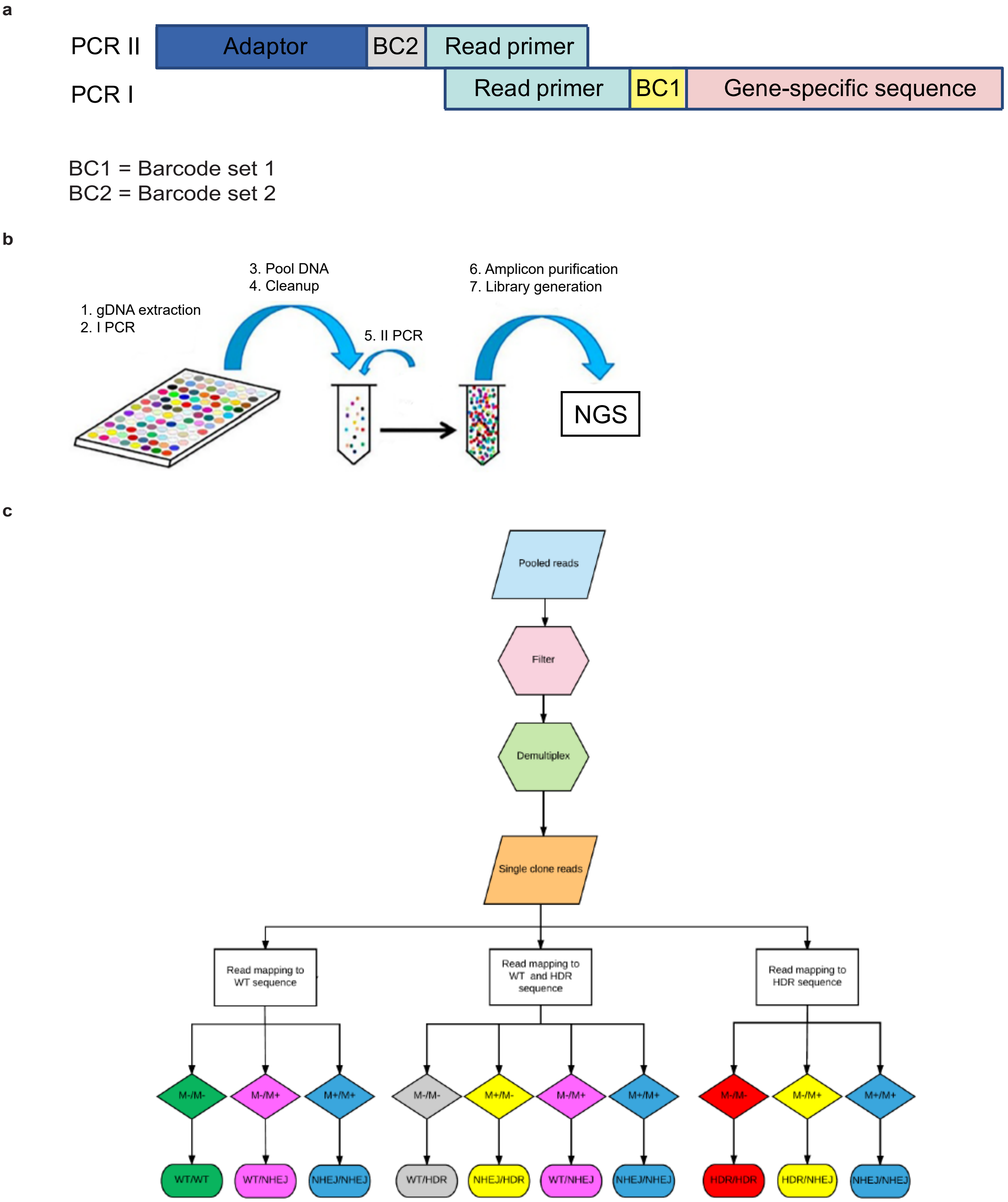

Supplementary Figure 2: Sample processing for NGS and data analysis for clone genotyping. **a** Organization of the sequencing primers used for NGS. The two-barcode system gives up to 96x96 index options using only two validated index sets. BC1=barcode set 1 (inline barcode, well identifier). BC2=barcode set 2 (plate identifier). **b** Schematic representation of the molecular biology workflow for genomic DNA extraction and sequencing library preparation from the exported clones (See Materials and Methods). **c** Flowchart of the NGS data analysis pipeline for genotype identification. After quality filtering and demultiplexing, reads from each clone were mapped to the reference WT or HDR sequence (M-). Clones presenting additional mismatches from the reference sequences (M+) in a 200bp region around the PAM site were identified as NHEJ.

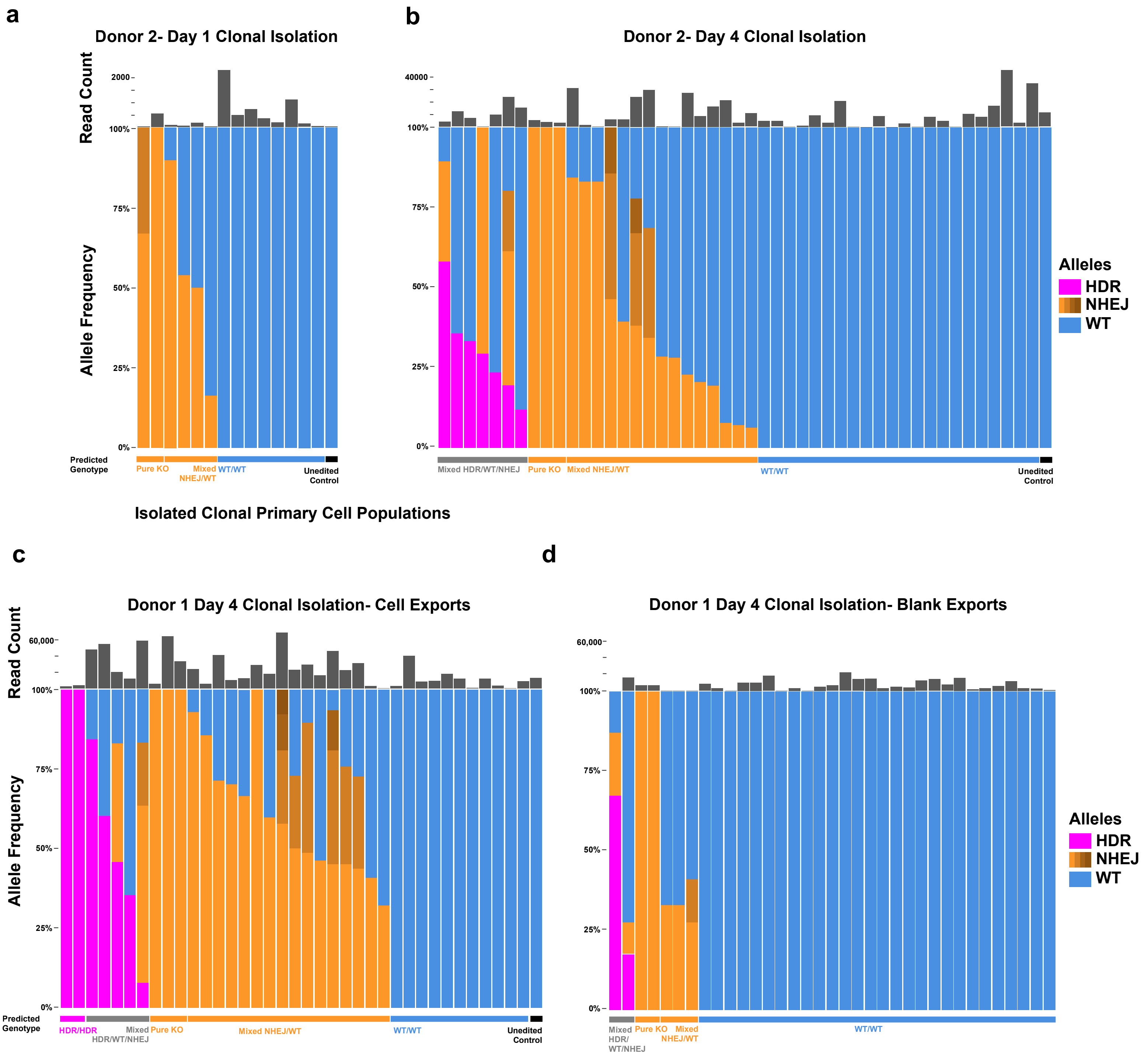

Supplementary Figure 3: Off-chip sequencing of clones from second healthy human donor and export controls. a-b Allele frequencies and read counts from cells isolated from a second healthy human blood donor and loaded on-chip either one day (**a**) or four days (**b**) after electroporation. **c-d** Cells were exported off-chip for sequencing (**c**) but as a control for cross-contamination introduced during the export procedure, media from NanoPens that had no cells loaded (Blank controls, **d**) was also exported off-chip and processed similarly for NGS sequencing. The presence of reads in these blank wells indicates that some cross-contamination between wells occurred, either on-chip, during the export of cells off the chip, or during the genomic PCR amplification or NGS library preparation steps after export. This potential cross-contamination also could contribute to the detection of more than two different alleles in some wells of the cell exports (**c**).

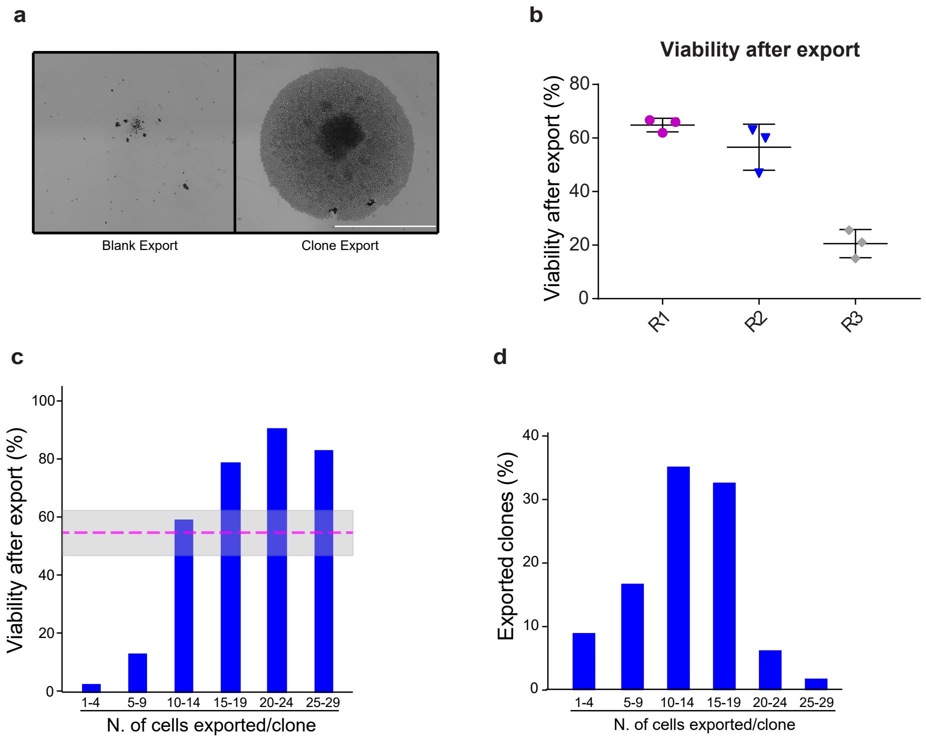

Supplementary Figure 4: Off-chip clonal expansion of exported clones. a Representative image of a colony after Culture export. Individual wells of the 96 well plates collected after export were imaged after 7 days in culture. Wells corresponding to blank exports were imaged as control **b** Dot plot of the viability after export in each experimental replicate (R1-3). Bars indicate means ±SD of three chips per each independent replicate (>20 clones exported per chip) **c** Graph, cell viability (% of clones forming colony) after export as a function of the number of cells exported. The magenta line and the gray area indicate, respectively, the mean and the SEM of cell viability across the exported clones (N=363 clones analyzed). **d** Quantification of the percentage of colonies exported as a function of the number of cells exported (N=363 clones analyzed).
