# Supplementary material for: Light-activated cell identification and sorting (LACIS) for selection of edited clones on a nanofluidic device
Source: Commun Biol. 2018 May 3;1:41. doi: 10.1038/s42003-018-0034-6 (PMC6123811; doi:10.1038/s42003-018-0034-6)
Supplement: Supplementary file 2 — Description of additional Supplementary Infomation [file 42003_2018_34_MOESM2_ESM.docx]

**Description of Additional Supplementary Items**

Supplementary Movie 1: Time-lapse images of single-cell selection, isolation and positioning of putatively edited T cells into individual NanoPen chambers using OptoSelect visible light technology. Images are acquired every 3 seconds in the Brightfield channel.

Supplementary Movie 2: Time-lapse images of single-cell selection, isolation and positioning of control T cells into individual NanoPen chambers using OptoSelect visible light technology. Images are acquired every 3 seconds in the Brightfield channel.

Supplementary Movie 3: Time-lapse of the culturing step of the split export process. Initially, the first half of a colony is unpenned using OEP (white bars), pushed into the channel, and exported into a well of a 96-well plate. The remaining cells are pushed back into the same NanoPen to maintain clonality.

Supplementary Movie 4: Time-lapse of the sequencing step of the split export process. The second half of the same colony is exported into a second 96-well plate for next generation sequencing and on-target validation.

Supplementary Data 1: The table describes the sequences of gRNA, and primers used in this study.
